# A Meta‐Analysis of Anti‐Thyroid Peroxidase Antibody and Omalizumab Response in Chronic Spontaneous Urticaria

**DOI:** 10.1111/cea.70363

**Published:** 2026-06-04

**Authors:** Ju Hyun Lee, Min Jung Geum, Do‐Young Kim, Young‐Mi Ah, Yun Mi Yu

**Affiliations:** ^1^ Graduate Program of Industrial Pharmaceutical Sciences Yonsei University Incheon Republic of Korea; ^2^ College of Pharmacy and Institute of Pharmaceutical Science and Technology Hanyang University Ansan Republic of Korea; ^3^ Department of Dermatology Yonsei University College of Medicine Seoul Korea; ^4^ College of Pharmacy Yeungnam University Gyeongsan Republic of Korea; ^5^ Department of Pharmacy and Yonsei Institute of Pharmaceutical Sciences, College of Pharmacy Yonsei University Incheon Republic of Korea; ^6^ Department of Pharmaceutical Medicine and Regulatory Science, Colleges of Medicine and Pharmacy Yonsei University Incheon Republic of Korea

## Abstract

- Treatment response to omalizumab in CSU varies, and no validated test reliably predicts responders.
- Anti‐TPO negative patients are more likely to respond to omalizumab; combined IgE may support clinical decision‐making.

To the Editor,

Chronic spontaneous urticaria (CSU) is a chronic immune‐mediated skin disorder characterized by the idiopathic occurrence of wheals, angioedema, or both for more than six weeks [1]. It affects approximately 0.1%–1.5% of the population and significantly reduces quality of life [2]. Second‐generation H1‐antihistamines are recommended as first‐line therapy; however, a subset of patients remains symptomatic and requires escalation to omalizumab, an anti‐immunoglobulin E (IgE) monoclonal antibody. Considering its high cost and highly variable treatment response, reliable predictive biomarkers are needed to ensure a more targeted therapy.

Thyroid autoimmunity has emerged as a possible factor influencing treatment response in CSU [3]. Anti‐thyroid peroxidase (anti‐TPO) antibodies are more frequently detected in patients with CSU and have been linked to an autoimmune subtype known as type IIb CSU that may be less responsive to omalizumab [3]. However, existing studies have shown conflicting results [4, 5, 6], and the predictive value of anti‐TPO status has not been systematically evaluated.

We conducted a systematic review and meta‐analysis to evaluate the association between thyroid autoimmunity and clinical response to omalizumab in patients with CSU, focusing on anti‐TPO antibody status and other relevant biomarkers, including IgE and C‐reactive protein (CRP). The MEDLINE, Embase, CENTRAL, SCOPUS, and Web of Science databases were systematically searched for relevant studies published from inception through March 1, 2026 (PROSPERO CRD420251148223). Eighteen cohort studies comprising 1846 patients were included. Detailed methods and additional analyses are available in the Open Science Framework (OSF) repository at https://doi.org/10.17605/OSF.IO/49VXA.

Patients without anti‐TPO positivity exhibited a significantly higher likelihood of responding to omalizumab compared to those with anti‐TPO positivity (OR 3.25, 95% CI 1.53–6.91). In subgroup analyses by region and income, no significant differences were observed between groups (*p* = 0.62 for region, *p* = 0.35 for income). Additionally, a significant association between anti‐TPO status and omalizumab response was confirmed in the group excluding low‐income countries (Table 1). Meta‐regression analyses consistently indicated that age, sex, disease duration, baseline CSU severity, and concomitant angioedema did not significantly influence the association. These findings remained robust in sensitivity analyses, including when treatment response was redefined as well‐controlled disease (OR 2.32, 95% CI 1.13–4.74) and after excluding a study with heterogeneous anti‐TPO cutoff (OR 4.18, 95% CI 2.13–8.18). However, no significant association was observed between baseline anti‐TPO levels and the response to omalizumab (MD –0.05 IU/mL, 95% CI –34.07 to 33.96). In pooled analyses of response rates including single‐arm studies, anti‐TPO–negative patients showed higher response rates than anti‐TPO–positive patients (87% vs. 72%); however, this difference was not significant. In the analyses considering IgE or CRP levels, patients with high IgE levels had a significantly greater likelihood of response (OR 5.34, 95% CI 2.94–9.69), whereas CRP status was not associated with treatment response (OR 1.10, 95% CI 0.57–2.12) (Table 1). Using the Grading of Recommendations, Assessment, Development, and Evaluation (GRADE) approach, the certainty of evidence for the association between anti‐TPO positivity and omalizumab response was rated low.

**TABLE 1:** Predictive value of baseline anti‐TPO, IgE and CRP for omalizumab response in patients with CSU.

Variables	No. of studies (patients)	OR (95% CI)^a^	*I* ^2^ (%)
*Anti‐TPO status*			
Negative vs. positive	9 (753)	3.25 (1.53–6.91)	47.8
Subgroup by geographic region^b^			
Asia	4 (237)	2.75 (1.25–6.06)	0
Non‐Asia	5 (516)	4.04 (1.08–15.05)	68.2
Subgroup by income level^b^			
High income	6 (591)	4.02 (1.38–11.66)	64.2
Low income	3 (162)	2.10 (0.90–4.91)	0
*IgE status*			
High versus low level	7 (641)	5.34 (2.94–9.69)	4.0
*CRP status*			
High versus normal/low level	4 (245)	1.10 (0.57–2.12)	0

Abbreviations: Anti‐TPO, anti‐thyroid peroxidase; CI, confidence interval; CRP, C‐reactive protein; CSU, chronic spontaneous urticaria; IgE, Immunoglobulin E; OR, odds ratio.

^a^ All pooled odds ratios and 95% CIs were calculated using the random‐effects model to account for potential between‐study heterogeneity.

^b^ The reference group for each analysis had an anti‐TPO positive status.

These findings may be interpreted in the context of CSU endotypes. CSU can be classified as types I (autoallergic, IgE‐associated) and IIb (autoimmune, IgG‐associated) [1]. Anti‐TPO positivity is associated with type IIb CSU, which is characterized by low total IgE levels and may be less responsive to omalizumab [1, 7]. Given that omalizumab exerts its therapeutic effect by binding free IgE and downregulating FcεRI expression on mast cells and basophils [8], the higher response rates observed in anti‐TPO–negative and IgE‐high patients are biologically plausible. These results suggest that anti‐TPO status, especially when coupled with total IgE, may serve as a surrogate marker of type IIb CSU and help inform clinical decision‐making regarding omalizumab treatment. The absence of an association between CRP positivity and treatment response is consistent with previous findings [9], likely reflecting the limited specificity of CRP for endotype‐specific pathophysiology. However, joint analyses incorporating both total IgE and anti‐TPO levels were not feasible owing to the lack of individual patient data in the included studies. Consequently, our findings should be interpreted with caution as a plausible hypothesis rather than a definitive conclusion.

This study has several strengths. To our knowledge, this is the first meta‐analysis to evaluate the association between anti‐TPO and response to omalizumab and highlights the potential of anti‐TPO as a biomarker. Rigorous methodological frameworks, including Quality in Prognostic Studies and GRADE, were applied to assess risk of bias and certainty of evidence, enhancing transparency and interpretability. Notably, we aligned our work with the tailored guide for prognostic factor research. The consistency of findings across sensitivity and subgroup analyses further supports the robustness of the results.

However, several limitations should be considered when interpreting these findings. Most included studies were observational with relatively small sample sizes, leading to low certainty of evidence and a susceptibility to residual confounding. Variability in response definitions and assessment timing may have affected comparability across studies. Furthermore, most studies reported anti‐TPO positivity rather than quantitative levels, limiting the ability to establish clinically relevant thresholds.

In conclusion, anti‐TPO–negative patients with CSU were more likely to respond to omalizumab than anti‐TPO–positive patients, with an approximately threefold higher likelihood of response. These findings suggest that thyroid autoimmunity may represent a surrogate marker of underlying immunologic features associated with differential responsiveness to omalizumab, particularly in combination with total IgE. Further large‐scale prospective studies are warranted to validate these results and investigate the association between the anti‐TPO/total IgE ratio and omalizumab responsiveness.

## Author Contributions

J.H.L. and M.J.G. contributed to the study design, data analysis and interpretation, and manuscript writing. D.‐Y.K. contributed to the review of the manuscript and data interpretation. Y.‐M.A. and Y.M.Y. contributed to the study conceptualization, interpretation of data, critical revision of the manuscript, and supervision of the study. All authors reviewed and approved the final version of the manuscript and agree to be accountable for all aspects of the work.

## Funding

This work was supported by the National Research Foundation of Korea (NRF) grant funded by the Korea government (MSIT) (No. RS‐2026‐25481749).

## Conflicts of Interest

The authors declare that the research was conducted in the absence of any commercial or financial relationships that could be construed as a potential conflicts of interest. J.H.L. is employed by Celltrion Inc.; however, this employment had no role in the study design, data collection, analysis, interpretation, or manuscript preparation, and no financial or non‐financial conflicts are associated with this work.

## Data Availability

All data generated or analyzed during this study are included in this published article and its supplementary materials, which are available in the Open Science Framework (OSF) repository at https://doi.org/10.17605/OSF.IO/49VXA.
